# Stress indicators based on airborne thermal imagery for field phenotyping a heterogeneous tree population for response to water constraints

**DOI:** 10.1093/jxb/eru309

**Published:** 2014-07-30

**Authors:** Nicolas Virlet, Valentine Lebourgeois, Sébastien Martinez, Evelyne Costes, Sylvain Labbé, Jean-Luc Regnard

**Affiliations:** ^1^Montpellier SupAgro, UMR 1334 Amélioration Génétique et Adaptation des Plantes méditerranéennes et tropicales, TA-A-108/03, Avenue Agropolis, 34398 Montpellier Cedex 5, France; ^2^CIRAD, UMR Territoires, Environnement, Télédétection et Information Spatiale, Station Ligne-Paradis, 7 Chemin de l’IRAT, 97410 Saint-Pierre, France; ^3^INRA, UMR 1334 AGAP, TA-A-108/03, Avenue Agropolis, Avenue Agropolis, 34398 Montpellier Cedex 5, France; ^4^IRSTEA, UMR TETIS, Remote Sensing Centre, 500 rue J. F. Breton, 34093 Montpellier Cedex 5, France

**Keywords:** Drought, evapotranspiration, *Malus × domestica* Borkh.,, phenomics, remote sensing, surface temperature, temperate fruit species, vegetation index, water deficit index (WDI).

## Abstract

As field phenotyping of plant response to water constraints constitutes a bottleneck for breeding programmes, airborne thermal imagery can contribute to assessing the water status of a wide range of individuals simultaneously. However, the presence of mixed soil–plant pixels in heterogeneous plant cover complicates the interpretation of canopy temperature. Moran’s Water Deficit Index (WDI = 1–ET_act_/ET_max_), which was designed to overcome this difficulty, was compared with surface minus air temperature (*T*_s_–*T*_a_) as a water stress indicator. As parameterization of the theoretical equations for WDI computation is difficult, particularly when applied to genotypes with large architectural variability, a simplified procedure based on quantile regression was proposed to delineate the Vegetation Index–Temperature (VIT) scatterplot. The sensitivity of WDI to variations in wet and dry references was assessed by applying more or less stringent quantile levels. The different stress indicators tested on a series of airborne multispectral images (RGB, near-infrared, and thermal infrared) of a population of 122 apple hybrids, under two irrigation regimes, significantly discriminated the tree water statuses. For each acquisition date, the statistical method efficiently delineated the VIT scatterplot, while the limits obtained using the theoretical approach overlapped it, leading to inconsistent WDI values. Once water constraint was established, the different stress indicators were linearly correlated to the stem water potential among a tree subset. *T*_s_–*T*_a_ showed a strong sensitivity to evaporative demand, which limited its relevancy for temporal comparisons. Finally, the statistical approach of WDI appeared the most suitable for high-throughput phenotyping.

## Highlight text

Thermal infrared imagery contributes to the phenotyping of crop response to water stress. Based on multispectral images, the Vegetation Index–Temperature (VIT) concept constitutes a relevant approach.

## Introduction

According to current climate change models for the 21st century, increases in average temperatures are expected, with longer or more frequent episodes of extreme temperatures and drought, notably in the Mediterranean basin (IPCC, 2007; Giorgi and Lionello, 2008). Climate change will lead to a reconsideration of breeding programmes for many crops, and new traits will need to be taken into account. As water is a major factor in plant productivity, optimizing water use by improving plant water use efficiency and/or stress tolerance will become an increasingly important issue for many crops (Hamdy *et al.*, 2003; Condon *et al.*, 2004). Breeding programmes focused on these targets are currently being developed for the cereal crops that are of major importance for food purposes, such as wheat, rice, and maize (Braun *et al.*, 2010; Fischer *et al.*, 2011; Masuka *et al.*, 2012). Such studies are rarely performed for woody perennials, although some studies on genetic determinism of traits related to water use and drought tolerance improvement have been undertaken for forest trees [e.g. in *Populus*, Dillen *et al.* (2008) and Monclus *et al.* (2009); in *Quercus*, Brendel *et al.* (2008) and Roussel *et al.* (2009)]. Water use and/or drought sensitivity in fruit trees also need to be thoroughly studied because sustainability of fruit production is highly dependent on the availability of water resources (Šircelj *et al.*, 2007). However, for these species, water resource research to date has focused on irrigation scheduling and crop management rather than on plant breeding for better use of water (Naor, 2006; Wang and Gartung, 2010).

Thermal infrared (TIR) remote sensing is increasingly being used to assess crop transpiration and water stress in annual crops and fruit crops (Berni *et al.*, 2009; Alchanatis *et al.*, 2010; González-Dugo *et al.*, 2012). As the temperature of any plant organ depends on the balance between incoming energy and energy loss, including the latent heat loss resulting from transpiration, measurement of leaf temperatures permits estimation of the flux of water loss and stomatal regulation (Jones *et al.*, 2009). Typically, an increase in the difference between the leaf surface temperature and the air temperature is interpreted as a sign of a decrease in transpiration flux, i.e. a decrease in the ratio of actual to potential transpiration. Nevertheless, as plant stomatal control can be more or less pronounced and can occur over the short term, the regulation of water loss by stomatal closure and the importance of transpiration limitation under water deficits depend on plant species. Tardieu and Simonneau (1998) classified plant behaviours into two main categories, isohydric and anisohydric, based on stomatal regulation. Facing water deficits, isohydric plants efficiently reduce stomatal conductance (*g*_sw_) in the presence of decreasing soil water potential (*Ψ*_s_) and/or drier atmospheric conditions, which contributes to saving water and maintaining a relatively constant midday leaf water potential (*Ψ*_l_) regardless of drought conditions (McDowell *et al.*, 2008). In contrast, anisohydric plants allow the midday *Ψ*_l_ to decrease with both higher evaporative demand and lower *Ψ*_s_ while maintaining a stomatal aperture, which favours carbon acquisition (McDowell *et al.*, 2008). As genetic variability of stomatal regulation can be expressed at the intraspecific level [e.g. in grapevine, Gaudillère *et al.* (2002); in apple, Massonnet *et al.* (2007)], TIR imagery appears to be a promising technique for phenotyping plant tolerance to water stress [in grapevine and rice, Jones *et al.* (2009); in potato, Prashar *et al.* (2013)]. More generally, phenotyping based on multi-spectral or hyper-spectral imagery shows promise as a non-invasive method of screening a wide range of individuals in a short period of time. This potentially high-throughput approach is compatible with next-generation sequencing technologies, which allow genome-wide and high-density genotyping (Berger *et al.*, 2010; Fiorani and Schurr, 2013).

Numerous indices have been developed to assess crop water stress from canopy surface temperature (*T*_s_) data acquired in signal or imagery mode, from aerial platforms (satellites, aircraft, and unmanned aerial vehicles) or sensors installed directly in fields to observe crop canopies (White *et al.*, 2012). *T*_s_ minus air temperature (*T*_a_) is a raw variable that is easy to extract from images, but it is sensitive to radiative conditions, wind speed, and vapour pressure deficit (Maes and Steppe, 2012). Temporal comparisons of plant responses to drought based on this variable require that ambient conditions are controlled (Berger *et al.*, 2010) or remain mostly unchanged during experiments (Idso *et al.*, 1981). The crop water stress index (CWSI) is one of the most commonly used indices in field water stress studies and irrigation scheduling applications. It takes into account the ambient meteorological conditions, including the vapour pressure deficit, which also influences the canopy temperature. CWSI was empirically developed by Idso *et al.* (1981) and theoretically defined by Jackson *et al.* (1981). With this index, the upper and lower limits of the differences in the canopy and air temperatures can be used to estimate the minimum and maximum evaporation and represent dry and wet references, respectively. Based on the CWSI concept, Jones *et al.* (2009) developed the stomatal conductance index (I_g_), which estimates stomatal conductance from the canopy temperature and some meteorological parameters, and these authors assessed the potential of TIR imaging to phenotype the response of crops to water constraints. However, the application of CWSI is limited to full-cover vegetation, and I_g_ requires measurements and calibrations (wet and dry references) at the leaf level, which may depend on genotype and limits their applications for high-throughput phenotyping.

The presence of mixed soil–plant pixels is a recurring problem when thermal imagery is applied to phenotyping heterogeneous covers (Möller *et al.*, 2007; Jones *et al.*, 2009; Hackl *et al.*, 2012). It is generally considered that using the vegetation surface temperature directly is risky, because the weight of mixed or soil pixels in porous plant cover can create a shift towards the soil surface temperature (Jackson *et al.*, 1981). Various image preprocessing methods based on filtering of RGB images can be applied to exclude mixed pixels. However, these methods are time-consuming and can also be subjective because they depend on the threshold chosen (Giuliani and Flore, 2000; Jones *et al.*, 2002; Möller *et al.*, 2007; Hackl *et al.*, 2012). An automated procedure that uses a watershed segmentation analysis to select pure vegetation pixels in TIR images of palm trees has recently been proposed by Cohen *et al.* (2012). However, as shown by Hackl *et al.* (2012), distinguishing pure pixels of vegetation from mixed soil–plant pixels does not always improve the quality of the regression between canopy temperature and plant water status indicators such as the leaf water potential in maize for cover fractions above 60%.

**Fig. 1.**
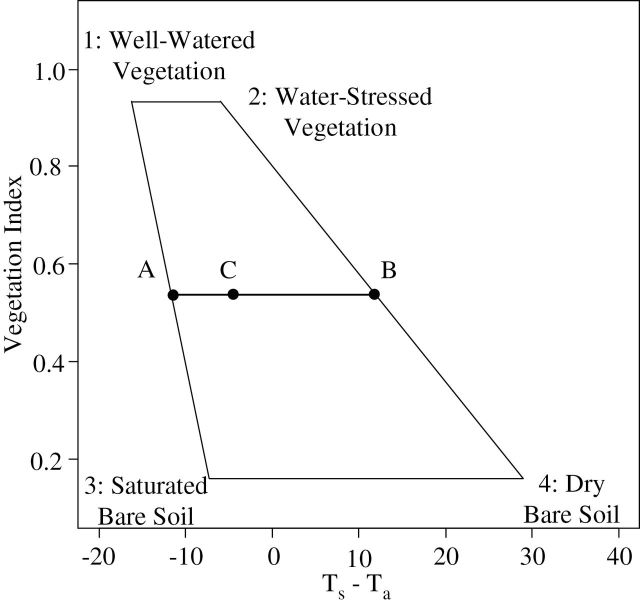
The hypothetical trapezoidal shape developed by Moran *et al.* (1994) resulting from the relationship between the plant surface temperature (*T*_*s*_) minus the air temperature (*T*_*a*_) and the vegetation index (NDVI or SAVI) used to represent the crop cover fraction. Vertices 1 to 4 represent extreme states of vegetation development and the evaporation/transpiration rate. For a given point C in the scatter plot, WDI is equal to the ratio between AC and AB. WDI values vary from 0 for well-watered conditions (fully transpiring vegetation) to 1 for water-stressed (no transpiration).

To overcome these limitations, Moran *et al.* (1994) developed the Vegetation Index–Temperature (VIT) concept, which facilitates the application of CWSI to partial vegetation cover. The VIT concept is based on the trapezoidal shape formed by the relationship between the difference between the surface and air temperature (*T*_s_–*T*_a_) and a vegetation index (VI) that represents the crop cover fraction. The vertices of the trapezoid correspond to (1) well-watered full-cover vegetation, (2) water-stressed full-cover vegetation, (3) saturated bare soil, and (4) dry bare soil, as shown in Fig. 1. Theoretically, all variations of crop water stress for different vegetation cover should plot within this trapezoid, even if linearity of the lateral boundaries constitutes a simplifying assumption (notably for low vegetation cover) (Maes and Steppe, 2012). For each point inside the trapezoid, the Water Deficit Index (WDI) can be calculated on the basis of the distances to the left and right boundaries, which are considered to be wet and dry references, respectively. By using this approach, the *T*_*s*_–*T*_*a*_ of a given plant is corrected from soil effects by VI according to the cover porosity. Like CWSI, WDI is related to the ratio of actual (ET_act_) to maximum (ET_max_) evapotranspiration (WDI = 1 – ET_act_ / ET_max_) and it takes the VPD into account. The value of WDI that is normalized between the left and right boundaries ranges from 0 for a well-watered crop transpiring at the maximum rate to 1 for a severely water-stressed, non-irrigated crop.

Theoretical calculation of the trapezoid envelope vertices requires information on numerous physical and meteorological parameters and knowledge of the vegetation type (Moran *et al.*, 1996). Consequently, this method is not easy to use operationally because of difficulties in its parameterization (Vidal and Devaux-Ros, 1995; Clarke, 1997) and because some inputs can be sensitive to local conditions or to the crop structure (Heilman *et al.*, 1996; Rana and Katerji, 1998). When using this approach for phenotyping, large variability in the plant architecture of the studied population may further complicate the parameterization of the theoretical equations. Moreover, the parameters’ values are generally typical values derived from the literature and not adapted to every type of crop or variety. The trapezoidal shape of the scatterplot can also be manually delineated based on VI and *T*_s_–*T*_a_ pixel values (Vidal and Devaux-Ros, 1995; Clarke, 1997). However, to be relevant, this empirical approach requires that TIR and vegetation index images encompass the extreme *T*_s_–*T*_a_ and VI values of dry/wet vegetation and soil. This requirement can be satisfied when working at a low spatial resolution with satellite images covering large areas, but a well-designed field set-up is necessary when imagery of higher spatial resolution (generally applied to smaller areas) is chosen, allowing each individual to be distinguished in phenotyping studies.

In this study, a statistical approach based on quantile regression is proposed to facilitate the delineation of the trapezoidal shape of the VIT scatterplot. This new approach is assumed to allow the application of WDI to field phenotyping of a tree population showing heterogeneous vegetation cover in response to water constraints. This statistical method as well as the theoretical method and the basic index, *T*_s_–*T*_a_, were tested on airborne multispectral images (RGB, near-infrared, and thermal infrared) of a population of 122 apple hybrids, characterized by their architectural variability (Segura *et al.*, 2008). The statistical and theoretical methods were compared with respect to their ability to delineate the VIT scatterplot under different environmental conditions, and their ease of implementation in the context of high-throughput phenotyping. The sensitivity of the WDI to changes in wet and dry references was tested by varying the levels of quantile within the statistical approach. The different stress indicators were compared in order to assess (i) their sensitivity in response to soil drought and (ii) their relevance to efficiently reflect the evolution of tree water status in comparison to the stem water potentials.

## Materials and methods

### Field experiments and meteorological measurements

**Fig. 2.**
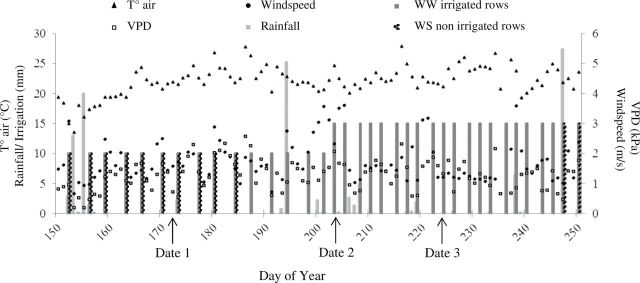
Meteorological and water management data relative to the field phenotyping experiment during the 2011 summer season. Black arrows represent the airborne image acquisition dates (dates 1, 2, 3). Air temperature, VPD and wind speed are averaged for each day.

The studied apple tree population consisted of progeny derived from a ‘Starkrimson’ × ‘Granny Smith’ cross and was characterized by strong variability in tree vigour, architectural traits (Segura *et al.*, 2008), biennial bearing (Guitton *et al.*, 2012), hydraulic traits (Lauri *et al.*, 2011), and stomatal and photosynthetic traits in response to vapour pressure deficit (Regnard *et al.*, 2009). In February 2007, four replicates of 122 hybrids and their two parents were grafted onto M9 rootstock and randomly distributed in an experimental field at the INRA Melgueil experimental station (in the southeast of France, N43°36, E03°58). The 520 trees were planted along 10 rows oriented northwest–southeast, with an inter-row spacing of 5 m and a 2 m spacing within the rows. A tall fescue (*Festuca arundinacea*) cover, 2.5 m wide, was sown in April 2007 in the inter-rows to create grass alleys. The grass was regularly mowed, and its border approached the vertical projection of the largest tree canopies. Within the tree rows, the soil was chemically weeded in the spring to prevent adventitious plant development. The field plot was irrigated using a system of microsprayers located in the rows, with one 20 l h^–1^ emitter per tree. A summer drought treatment was applied on two repetitions per genotype beginning on 8 July (corresponding to day of year, DOY, 189) to 5 rows out of 10, resulting in progressive water stress (WS) for these trees because the summer rainfall was very limited (Fig. 2). The five WS rows were alternated with five other rows of trees that were well watered (WW). Irrigation of the WW trees was scheduled on the basis of the soil water potential (*ψ*_*s*_ at 30cm and 60cm depths) measured in a representative area of the field. It was performed twice a week from DOY 131 to 203 using an amount of water corresponding to ~3mm per day and then three times a week from DOY 204 onward with an amount of water corresponding to ~6.5mm per day (Fig. 2).

The trees were left unpruned during the entire experiment. Control of pests and diseases, tree fertilization, and fruit thinning were performed by conventional means, consistent with professional practices, throughout the study. Automated soil resistivity profiling conducted in March 2009 showed that the soil of the trial plot (at depths of 0–50cm and 50–100cm) could be considered spatially homogeneous in terms of its water-holding capacity.

The environmental conditions were monitored using meteorological sensors that recorded the global radiation (*R*_*g*_), air temperature (*T*_*a*_), air humidity (*HR*), air vapour pressure deficit (*VPD*), wind speed (*u*) and rainfall. These data were scanned every 30 s, averaged over 10-min intervals, and stored using a Campbell Scientific CR10X data logger. During the airborne image acquisition, the data collected were averaged over 2min intervals. Table 1 presents the values of *R*_*g*_, *T*_*a*_, *HR*, and *u*, averaged over the 12-min image acquisition periods during three flights, the corresponding *VPD* values, and the average values of *ψ*_*s*_ measured for six WW trees and six WS trees at 30- and 60-cm depths just before the flights.

**Table 1.** Environmental conditions for each image acquisition date^a^

Variable	Units	Date 1	Date 2	Date 3
Solar time	hh:mm	10:00	10:00	09:20
R_g_	W m^–2^	770.67 (3.27)	599.27 (102.85)	705.00 (0.00)
T°_air_	°C	26.91 (0.19)	26.58 (0.33)	26.85 (0.49)
RH	%	58.72 (0.75)	27.96 (0.33)	31.80 (1.86)
VPD	kPa	1.47 (0.04)	2.51 (0.06)	2.41 (0.14)
u	m s^–1^	1.99 (0.36)	1.73 (0.28)	0.78 (0.32)
Ψ_s_ WW	MPa	–0.022 (0.012)	–0.046 (0.039)	–0.024 (0.036)
Ψ_s_ WS	MPa	–0.031 (0.021)	–0.078 (0.037)	–0.130 (0.048)

^a^ Atmospheric values are means (and SD) over intervals of 12min, corresponding to three periods of flight over the experimental plot. ψs values are average measurements taken at soil depths of 30 and 60cm for six WW trees and six WS trees just before the flights.

### Image acquisitions

The image acquisition system consisted of an ultra-light aircraft equipped with two EOS 500D (Canon^®^, Tokyo, Japan) commercial digital cameras (15.1 Megapixel CMOS sensor) with 35-mm lenses and one FLIR B20HSV (FLIR Systems Inc., Wilsonville, USA) thermal infrared camera (320×240 matrix). The spectral sensitivity of the two digital cameras was measured in the laboratory with a monochromatic source 1.2nm wide (Déliot *et al.*, 2005). One camera acquired visible images in the red, green, and blue (RGB) bands. The second was modified according to Lebourgeois *et al.* (2008, 2012) to obtain images in the near-infrared band (NIR). The settings of the two cameras (aperture, F3.5; shutter speed, 1/2000; and sensitivity, ISO100) remained unchanged throughout the experiment, except on the first acquisition date (shutter speed of the NIR camera: 1/2500). Images were acquired in raw format and, after correction of vignetting effects, digital numbers (DN) for each spectral band were retrieved by decoding the images, as described in Lebourgeois *et al.* (2008). For the TIR camera, the radiation detected over the spectral range of 7.5–13 µm was considered equivalent to the surface temperature, assuming a target emissivity equal to unity. As the TIR images were acquired 2h before solar noon with a narrow-angle lens (72mm) and a vertical view, the effects of shadows and directional radiance were minimized (Jones *et al.*, 2009). The TIR images acquired had a radiometric resolution of 0.1°C and an absolute precision of 2°C. All cameras pointed in the same vertical direction, and their shutters were synchronized to a single trigger.

Three flights were performed during the summer of 2011, on DOY 172 (date 1, 17 days before the beginning of the drought treatment), 203, and 223 (dates 2 and 3, and 14 and 34 days, respectively, after the beginning of the drought treatment). Consequently, on the first acquisition date, the WS trees had not yet been subjected to water constraint. The image acquisitions took place in the early phase of stomatal regulation, at approximately 10:00 GMT, early enough to prevent atmospheric disturbance caused by thermal wind that frequently arises after this hour. The images were acquired at an elevation of 300 m and had a 3cm resolution in RGB and NIR, and a resolution of 30cm in TIR (40cm for the third acquisition date). At this altitude, the orchard (100×60 m) was covered by 1 or 2 images.

**Fig. 3.**
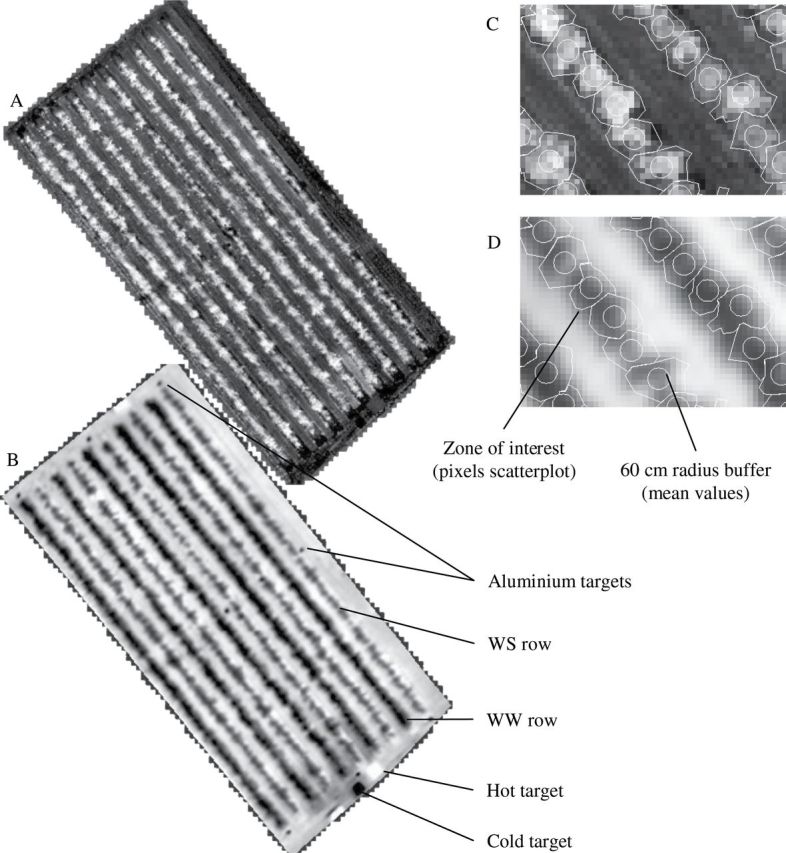
NDVI (A, C) and *T*_*s*_–*T*_*a*_ (B, D) images of the apple orchard on date 2. Zones of interest, larger than individual tree canopies, were manually drawn and merged to compute the field study mask, with delineation based on NDVI images (C) and then applied to *T*_*s*_–*T*_*a*_ images (D). For each individual tree, a 60-cm-radius buffer (representative zone) was located at the tree centre.

Nine aluminium targets were distributed around the periphery and within the experimental field for image geolocation (Fig. 3). A differential Global Positioning System (DGPS) was used to determine the precise position of each target, which was easily located in the thermal images due to the low emissivity of aluminium. Ground surface temperatures were acquired simultaneously for a cold target (Styrofoam: 2×2 m), bare soil, and a hot target (dark plastic cover: 4×4 m) via airborne acquisition to correct for atmospheric effects on the airborne TIR images. Temperature measurements at the ground level in the field were performed using a Fluke 574 hand-held infrared thermometer (Fluke Corporation, Everett, USA) with a resolution of 0.1°C and a precision of 0.75°C.

### Measurement of stem water potential

Stem water potential was chosen for use as a measure of the trees’ water status (Doltra *et al.*, 2007). Measurements of stem water potential (*ψ*_*H*_ *stem*) were performed using a pressure chamber (Model 3005 Soilmoisture Equipment Corp., Santa Barbara, USA) on 12 trees on dates 1 and 2 and on 20 trees on date 3. Shaded leaves located near the trunk were selected and confined in a plastic bag covered by aluminium foil for 2h before the measurement to stop transpiration and eliminate any water potential gradient between the leaf, stem, and branches (Goldhamer and Fereres, 2001). Pressure chamber measurements began immediately after the airborne image acquisitions, and required 1–1.5h to accomplish.

### Spectral image preprocessing and vegetation index computation

The image preprocessing operations were performed with Erdas Imagine 9.3 software (Intergraph Corporation, Huntsville, USA). A vignetting correction was applied to the NIR images according to the method described by Lebourgeois *et al.* (2008). Geometric distortions in the RGB and NIR images were corrected using orthorectification based on a second-order polynomial model and the geo-located aluminium targets as references. To correct the effects of (i) differences in solar radiation on the different dates and (ii) a quicker shutter speed on date 1 for the NIR camera, a radiometric normalization based on invariant field targets was performed for dates 1 and 3 in each spectral band, with date 2 as a reference, so that the acquisitions would be comparable from one date to another.

After orthorectification based on both the RGB and NIR images and the geo-located aluminium targets, the TIR images were corrected for atmospheric effects using ground surface temperatures acquired for the bare soil and hot and cold targets, as described previously. *T*_s_–*T*_a_ images were obtained by subtracting the air temperature acquired simultaneously from each pixel value of the TIR images.

The normalized difference vegetation index (NDVI; Rouse *et al.*, 1973) was used to estimate the crop cover fraction and was calculated as follows: NDVI = (NIR – R) / (NIR + R), where R and NIR are the digital values recorded by the cameras in the red and near-infrared bands, respectively. Bidirectional reflectance distribution heterogeneity was generally assumed to be effectively normalized through band ratio indices (Colwell, 1974). In our case, the images were acquired at 10:00 GMT (high sun elevation), with a limited lens field of view (35mm); thus, the calculated NDVI was assumed to limit most of these directional effects. As NIR and R corresponded to DN, the range of NDVI values obtained in this study did not correspond well to the typical values mentioned in the literature for NIR and R that correspond to reflectance values. The spatial resolution of the NDVI images was degraded from 3 to 30cm to match the TIR image resolution on dates 1 and 2 and from 3 to 40cm to match the TIR image resolution on date 3.

For each of the 520 trees, a zone of interest larger than the crown, common to each of the three dates and containing balanced proportions of ground and tree vegetation, was manually drawn to serve as a study mask (Fig. 3). This study mask was used to avoid the influence of the distribution of the scatterplot in the determination of the left and right envelopes, using a statistical method to limit the weight of dry grass or bare soil pixels for the inter-rows. After merging the 520 zones, the pixel values of NDVI and *T*_s_–*T*_a_ were extracted from the resulting mask of the whole field plot and used to obtain the VIT scatterplot for each date. Representative NDVI and *T*_s_–*T*_a_ values were also extracted for each individual tree from a circular buffer with a 60-cm radius around the centre of the tree. Given the 30 or 40cm resolution of the images, each buffer contained 12–16 pixels. For each tree, mean NDVI and *T*_s_–*T*_a_ were used to compute statistical and theoretical WDI values (hereafter referred to as WDI_qr and WDI_t, respectively) for each tree and each date.

### Theoretical approach to WDI calculation

In the theoretical VIT approach developed by Moran *et al.* (1994), vertices of the trapezoid constitute four extreme states of vegetation cover and water status, as described previously (Fig. 1). WDI is computed as the ratio of distances AC/AB, where AC and AB correspond to [(*T*_*s*_ *–T*_*a*_) – (*T*_*s*_ *–T*_*a_min*_)] and [(*T*_*s*_ *–T*_*a_max*_) *–* (*T*_*s*_ *–T*_*a_min*_)], respectively. (*T*_*s*_ *–T*_*a_min*_) and (*T*_*s*_ *–T*_*a_max*_) represent the wet and dry references, respectively, of WDI. These temperature references depend on the NDVI value (Fig. 1). For each vertex, the (*T*_*s*_ *–T*_*a*_)_*i*_ value (the subscript *i* refers to vertices 1 to 4 in Fig. 1) is based on physical energy balance equations and can be computed as follows:

For well-watered dense vegetation:

$${\left(T_{s}-T_{a}\right)}_{1}=\frac{r_{a}\left(R_{n}-G\right)}{C_{v}}\times \frac{\gamma \left(1+\frac{r_{cp}}{r_{a}}\right)}{\Delta +\gamma \left(1+\frac{r_{cp}}{r_{a}}\right)}-\left(\frac{VPD}{\Delta +\gamma \left(1+\frac{r_{cp}}{r_{a}}\right)}\right)$$ (1)

where *r*_*a*_ is the aerodynamic resistance (s m^–1^), *R*_*n*_ is the net radiation (W m^–2^), *G* is the soil heat flux density (W m^–2^), *C*_*v*_ is the volumetric heat capacity of air (J °C^–1^ m^–3^), *γ* is the psychrometric constant (kPa °C^–1^), *r*_*cp*_ and *r*_*cx*_ are the canopy resistances to vapour transfer for fully transpiring and fully stressed cover, respectively (s m^–1^), *VPD* is the air vapour pressure deficit (kPa), and *Δ* is the slope of the saturated vapour pressure–temperature relationship (kPa °C^–1^). The parameters *γ* and *Δ* are computed from equations in the FAO 56 database.

For water-stressed dense vegetation:

$${\left(T_{s}-T_{a}\right)}_{2}=\frac{r_{a}\left(R_{n}-G\right)}{C_{v}}\times \frac{\gamma \left(1+\frac{r_{cx}}{r_{a}}\right)}{\Delta +\gamma \left(1+\frac{r_{cx}}{r_{a}}\right)}-\left(\frac{VPD}{\Delta +\gamma \left(1+\frac{r_{cx}}{r_{a}}\right)}\right)$$ (2)

For water-saturated bare soil, where canopy resistance r_c_ = 0:

$${\left(T_{s}-T_{a}\right)}_{3}=\frac{r_{a}\left(R_{n}-G\right)}{C_{v}}\times \frac{\gamma }{\Delta +\gamma }-\left(\frac{VPD}{\Delta +\gamma }\right)$$ (3)

and for dry bare soil, where r_c_ = ∞:

$${\left(T_{s}-T_{a}\right)}_{4}=\frac{r_{a}\left(R_{n}-G\right)}{C_{v}}$$ (4)

The net radiation *R*_*n*_ was computed from the classical expression for the surface radiation budget:

$$R_{n}=\left(1-\alpha \right)\times R_{g}+R_{a}-\varepsilon \sigma T_{s}^{4}$$ (5)

In eqn 5, *α* is the surface albedo [assumed to be equal to 0.18, 0.16, and 0.24 for vegetation, saturated bare soil, and dry bare soil, respectively, according to Bsaibes *et al.* (2009)], *R*_*g*_ is the incoming shortwave solar radiation (W m^–2^), *R*_*a*_ is the atmospheric radiation, computed as described by Brutsaert (1975) and equal to 0.83 for our conditions, *ε* is the surface emissivity (0.98, 0.95, and 0.92 for vegetation, saturated bare soil, and dry bare soil, respectively; Fuchs and Tanner, 1968; Mira *et al.*, 2007), *σ* is the Stefan–Boltzmann constant (5.67 10^–8^ W m^–2^ K^–4^) and *T*_*s*_ is the radiative surface temperature (K). The soil heat flux density *G* was computed from *R*_*n*_ with *G* equal to 0.15 × *R*_*n*_ for vegetation, 0.3 × *R*_*n*_ for saturated bare soil and 0.5 × *R*_*n*_ for dry bare soil (Idso *et al.*, 1975).

The aerodynamic resistance *r*_*a*_ in eqns 1 to 4 was computed as defined by Thom and Oliver (1977). The equation proposed by these authors is well suited to low-wind speed conditions (Jackson *et al.*, 1988) and was therefore adopted for use in this study because the measured wind speed *u* was always less than 2 m s^–1^ during the image acquisitions (see Table 1):

$$r_{a}=4.72\times \frac{{\left(\ln\left(\frac{z-d}{z_{0}}\right)\right)}^{2}}{1+0.54u}$$ (6)

In eqn 6, *z* is the anemometer height (3.2 m), *u* is the wind speed (m s^–1^), *d* is the zero-plane displacement (e.g. for apple trees, 2.49 m; Mihailović *et al.*, 2010), *z*_*0*_ is the roughness parameter (e.g. for apple trees, 0.24 m; Mihailović *et al.*, 2010), and *h* is the maximum vegetation height (3.5 m). The values *r*_*cp*_ and *r*_*cx*_ depend on stomatal closure and can be determined from the stomatal resistance (*r*_*s*_) and the maximum leaf area index (LAI): *r*_*c*_ = *r*_*s*_ / *LAI* (Moran *et al.*, 1994). A value of 4 was assumed for the maximum LAI. The minimum stomatal resistance in the WW condition (*r*_*cp*_) was taken to be 38 s m^–1^ [cv. Granny Smith in Liu *et al.* (2012)] and the maximum value was taken to be 1171 s m^–1^ in the WS condition (*r*_*cx*_) (team data, unpublished).

### Statistical approach to WDI calculation

Quantile regression is a method for describing the relations between variables for all portions of a probability distribution (Koenker and Bassett, 1978). This method was used in this study in order to easily define wet and dry references of the trapezoid envelope of *T*_s_–*T*_a_ vs NDVI for each acquisition date. The left and right boundaries (oblique lines 1–3 and 2–4 corresponding to the wet and dry references, respectively, as shown in Fig. 1) were obtained by calculating the quantile regression of *T*_s_–T_a_ as a function of NDVI. We applied six quantile levels for the left (0.1, 0.5, 1, 2, 3, and 4%) and right (99.9, 99.5, 99, 98, 97, and 96%) boundaries, thereby delimiting 99.8, 99, 98, 96, 94, and 92% of the scatterplot. The horizontal upper and lower limits of the scatterplot were determined from the maximum and minimum observed values of NDVI. WDI values were calculated for each pair of left and right boundaries obtained from the different quantiles and designated as WDI_n (n representing the interval percentage). The calculations were carried out using the quantreg software package (R Development Core Team 2008), developed by Koenker (2008).

### Statistical analysis

Analyses were performed for 464 trees, i.e. for 116 genotypes replicated four times. Six genotypes, for which one of the four replicates died, were not taken into account. All the experimental data were analysed with the R software v.2.13.2. After normality and homoscedasticity tests, analyses of variance (ANOVA) were performed to test (i) the effect of WDI computation method on responses of WW and WS trees, (ii) the drought effect at each date for NDVI and the different water stress indicators, and (iii) the date effect on WW and WS trees for the same variables. The quality of the linear regressions between *T*_s_–*T*_a_, WDI_qr and WDI_t and the plant stem water potential was assessed using the coefficient of determination (*R* ^2^) for each date and all dates confounded.

## Results

### VIT trapezoid envelope computation

**Fig. 4.**
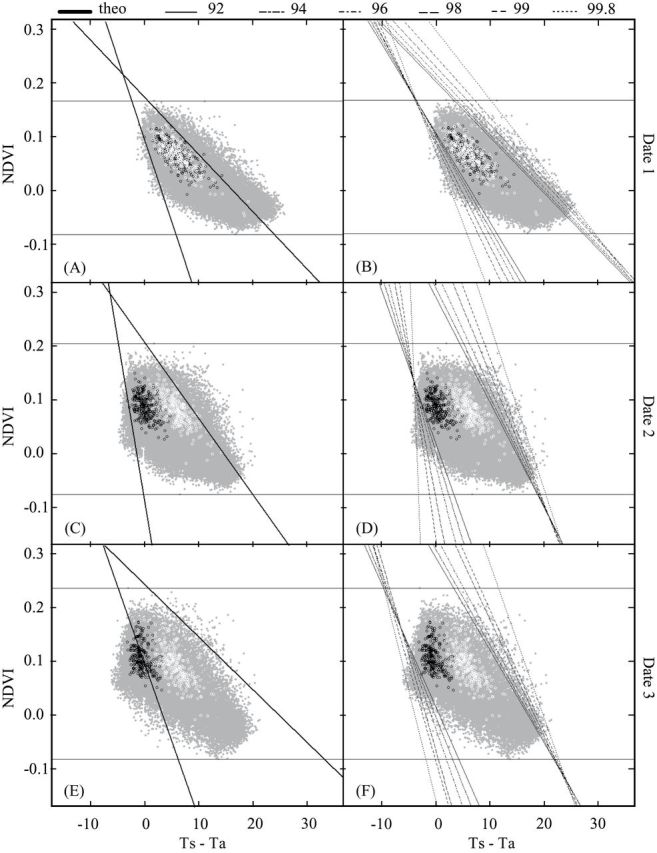
Envelopes of VIT trapezoids obtained using the theoretical approach (A, C, and E) and quantile regression (B, D, and F) for the three flights over the field plot on dates 1 (A, B), 2 (C, D), and 3 (E, F). Grey points represent pixel values inside the study mask for NDVI (y-axis) and *T*_*s*_–*T*_*a*_ (x-axis). Black and white circles represent the average y/x values for individual WW and WS trees, respectively.

Plots of NDVI versus *T*_s_–*T*_a_ for the three dates are presented in Fig. 4 for each pixel of the study mask (VIT scatterplot: grey points). The theoretical method was applied to delineate the VIT scatterplot for the three image acquisition dates (Fig. 4 A, C, and E, respectively). The left boundary correctly delineated the scatterplot (grey points) for dates 1 and 2 only (Fig. 4A and C). In contrast, the left boundary overlapped the scatterplot for date 3 (Fig. 4E), excluding approximately one quarter of the scatterplot. The right boundary delineated the right part of the scatterplot satisfactorily for date 3 (Fig. 4E) but overlapped it for dates 1 and 2 (Fig. 4A and C). The theoretical left and/or right boundaries did not satisfactorily delineate the scatterplot for any of the acquisition dates. However, for dates 1 and 2, the majority of the points representing the average values of NDVI and *T*_s_–*T*_a_ per tree (white and black circles) were included in the trapezoid. In contrast, for date 3, a number of the average values of NDVI and *T*_s_–*T*_a_ for the WW trees were located outside the left border of the theoretical envelope, leading to out-of-range WDI values (see below).

With the statistical method (Fig. 4B, D, and F), various trapezoid limits were obtained depending on the quantile levels used. Lines delimiting the left and right boundaries overlapped the scatterplot slightly, increasingly as the percentage of the scatterplot used in the quantile regression decreased. However, for each date, the computed boundaries delimited all the average values of NDVI and *T*_s_–*T*_a_ per tree. The left boundaries obtained with the different quantile levels used in the regression converged at the upper left part of the scatterplot (corresponding to vertex 1; see Fig. 1) while the right boundaries diverged at the upper right part of the scatterplot for each date (vertex 2, Fig. 1). The values for the WW trees were mostly located in the upper left part of the scatterplot, near the convergence point (Fig. 4B, D, and F). In contrast, the values for the WS trees were located in the upper right half of the scatterplot, near the different right boundaries whose divergences could affect the WDI values of the WS trees. When different water regimes were established, on dates 2 and 3, data points corresponding to WW and WS trees appeared clearly separated.

### Influence of left and right boundaries

**Fig. 5.**
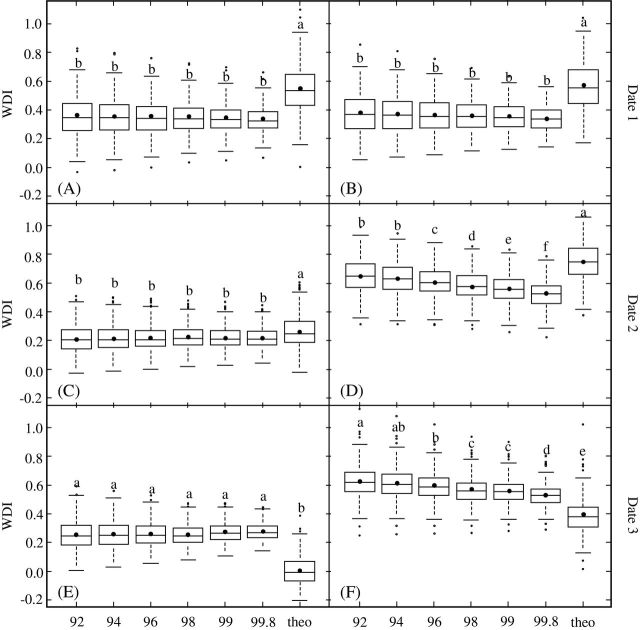
Distribution of WDI values (116 genotypes × 2 replicates) for the theoretical (theo) and statistical (quantile regression using 92–99.8% of scatter plot) methods for the three dates: dates 1 (A, B), 2 (C, D), and 3 (E, F), and for the two irrigation regimes, WW and WS. Each box plot shows the median value, the 1st and 3rd quartiles, and the 1st and 9th deciles. Mean values of WDI are represented by closed black circles inside box plots. For each irrigation regime and each date, lower-case letters identify mean WDI values that are significantly different from each other (NK test; α = 0.05).

Influence of the trapezoid boundaries, obtained using the statistical method, on WDI distribution and mean values was studied for each date for the WW and WS trees using boxplots (Fig. 5A, C, and E; and Fig. 5B, D, and F, respectively). Regardless of the unsatisfactory results obtained using theoretical trapezoid delineation, the WDI_t values obtained were also included in these comparisons.

The ranges of tree WDI values obtained by the theoretical method (WDI_t) were larger than the ranges obtained from the different quantile regressions for date 1 (Fig. 5A and B) and were comparable to the ranges of WDI_92 values for dates 2 and 3 (Fig. 5C, D, E, and F). The means of WDI_t values were significantly higher than those of the different WDI_qr values for dates 1 and 2, whatever the water regime (Fig. 5A, B, C, and D), and lower for date 3 (Fig. 5E and F). The low and even negative values of WDI_t obtained in WW trees for date 3 can be explained by the overlapping of the left theoretical boundary on a portion of the points corresponding to the WW trees (Fig. 4E). The mean WDI_t presented inconsistent values regardless of the water treatment applied: the mean values obtained on date 1 for the WW and WS trees (0.55 and 0.57), before water stress, were unexpectedly greater than that for the WS trees (0.38) on date 3 (when water stress was well established).

The range of statistical WDI values computed from the different quantile regression (WDI_qr) for each date decreased as the percentage of the scatterplot taken into account increased, for both the WW and the WS trees. When only the mean WDI_qr value of each quantile was considered, no significant differences were observed for the WW trees for any of the three dates (Fig. 5A, C, and E) or for WS trees for the well-watered conditions on date 1 (Fig. 5B). In contrast, on dates 2 and 3, the mean WDI_qr values of the WS trees decreased significantly when the percentage of the scatterplot increased (Fig. 4D and F). It remained nevertheless higher than in WW trees.

### Influence of environmental conditions: drought and date effects

The effects of irrigation regime and observation date on WW and WS trees are presented in Table 2, considering different phenotypic variables. As these effects were similar on all the WDI_qr values, only the WDI_qr computed for the maximum and minimum quantile used are presented.

**Table 2.** ANOVA testing the soil drought and date effects for WW and WS trees on NDVI, T_s_–T_a_, WDI_92, WDI_99.8, and WDI_t variables*

		NDVI	Ts–Ta	WDI_92	WDI_99.8	WDI_t
Date 1	WW	0.072 (0.023)^c^	6.05 (1.87)^a^	0.36 (0.11)^a^	0.34 (0.07)^a^	0.55 (0.13)^a^
	WS	0.074 (0.025)^C^	6.08 (1.84)	0.37 (0.09)^C^	0.34 (0.06)^B^	0.57 (0.11)^B^
	**Drought effect**	ns	ns	ns	ns	ns
Date 2	WW	0.090 (0.022)^b^	0.38 (1.00)^b^	0.22 (0.07)^c^	0.22 (0.05)^c^	0.27 (0.08)^b^
	WS	0.087 (0.027)^B^	6.01 (1.67)	0.65 (0.09)^A^	0.52 (0.07)^A^	0.75 (0.10)^A^
	**Drought effect**	ns	***	***	***	***
Date 3	WW	0.111 (0.024)^a^	–0.37 (0.92)^c^	0.25 (0.08)^b^	0.27 (0.04)^b^	0.00 (0.07)^c^
	WS	0.098 (0.031)^A^	5.81 (1.54)	0.62 (0.09)^B^	0.52 (0.05)^A^	0.38 (0.10)^C^
	**Drought effect**	***	***	***	***	***
*Date effect*	WW	***	***	***	***	***
	WS	***	ns	***	***	***

* Values are means (and SD) for the water regimes for each date. Significant *P*-values are represented as follows: * for *P* ≤ 0.05, ** for *P* ≤ 0.01 and *** for *P* ≤ 0.001; ns, not significant. According to the post*-*hoc Student-Newman-Keuls test (α = 0.05) different letters indicate when significant differences exist between dates for each variable, lower-case and upper-case letters being relative to WW and WS trees, respectively.

The drought effect was tested considering the different experiment dates separately. For date 1 (DOY 172), where irrigation regime in WW and WS tree was not differentiated (see *ψ*_*s*_ in Table 1), no significant differences were observed for any of the variables. The effect of drought was significant only for date 3 for NDVI with lower value for WS trees. The drought effect was significant (*P* ≤ 0.001) for dates 2 and 3, with an increase in the values of each variable related to water status, *T*_s_–*T*_a_, WDI_qr, and WDI_t.

The date effect is analysed thereafter for WW and WS trees separately (Table 2). For NDVI, a date effect was observed, with a significant increase of mean values from date 1 to date 3, whatever the water regime. The temporal increase of NDVI values between these dates was slightly less in WS trees (+0.024) than in WW trees (+0.039). Concerning *T*_s_–*T*_a_, the mean value observed for date 1 for WW trees (about 6°C) was more elevated than those observed on the same trees for dates 2 and 3 (nearing 0°, Table 2). *T*_s_–*T*_a_ mean values of WS trees for dates 2 and 3, where WS trees were submitted to soil drought, presented similar and non-significantly different values with date 1 (around 6°C), where WS trees were still irrigated.

A significant date effect was observed for the different WDI computation methods (Table 2). For WW trees, significant variations were observed, the lowest WDI values being observed for date 2 (0.22). For WS trees, a significant increase of mean WDI_qr was observed on dates 2 and 3 compared to date 1. For WDI_92, mean value was slightly but significantly higher for date 2 than for date 3 (0.65 and 0.62, respectively) while the WDI_99.8 mean values were equal for dates 2 and 3. WDI_t also presented a date effect for WW and WS trees. As stated previously in Fig. 4, this variable showed meaningless variations. Mean WDI_t decreased significantly in the WW trees, from date 1 to date 3, while it was significantly higher for date 2 in the WS trees. Mean WDI_t value of WS trees for date 3 was significantly lower than the value obtained for date 1, where WS trees were still well-irrigated.

### Image-based stress indicators versus stem water potential (*ψ* *H* *stem*)

**Fig. 6.**
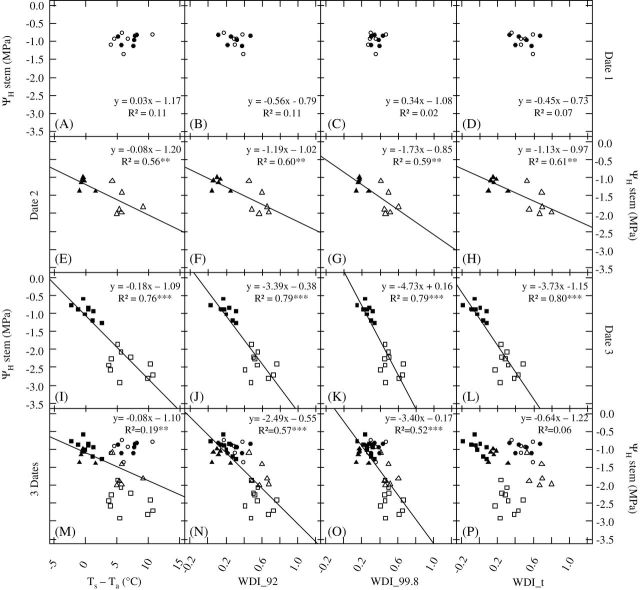
Linear regressions between stem water potential (*ψ*_*H*_ *stem*) and *T*_*s*_–*T*_*a*_ (A, E, I, and M), WDI_92 (B, F, J, and N), WDI_99.8 (C, G, K, and O) and WDI_t (D, H, L, and P) for the three flight dates separately (date 1, A, B, C, and D; date 2, E, F, G, and H; date 3, I, J, K, and L), or confounded (M, N, O, and P). Closed symbols correspond to WW trees and open symbols correspond to WS trees. Circles, triangles, and squares correspond to dates 1, 2, and 3, respectively. Significant regression *P*-values are represented as follows: * for *P* ≤ 0.05, ** for *P* ≤ 0.01, and *** for *P* ≤ 0.001.

Linear regressions of *ψ*_*H*_ *stem* against *T*_s_–*T*_a_, WDI_qr, and WDI_t were established for a subset of 12 trees for date 1 (all well watered), six WW and six WS trees for date 2, and 10 WW and 10 WS trees for date 3 (Fig. 6). For date 1 (Fig. 6A to D), *T*_s_–*T*_a_, WDI_qr, and WDI_t were not correlated with *ψ*_*H*_ *stem*, whose range of variation was very limited (–0.70 to –1.35MPa) because WS trees were not yet submitted to water stress. Linear regressions between *ψ*_*H*_ *stem* and *T*_s_–*T*_a_ showed significant negative correlations once water constraint was established on the WS population (for dates 2 and 3; Fig. 6E and I). Similarly, on the same dates, WDI_qr and WDI_t were significantly correlated with *ψ*_*H*__*stem*_ (Fig. 6F, G, and H and Fig. 6J, K, and L) regardless of the computation method considered. For date 2 (Fig. 6E to H), i.e. for conditions of moderate water constraint, the *R* ^2^ values corresponding to the regressions between *ψ*_*H*__*stem*_ values and those of image-based indicators were significant (*P* ≤ 0.01) and ranged between 0.56 and 0.61. For date 3 (Fig. 6I to L), i.e. for conditions of more severe water constraint, leading to a larger range of *ψ*_*H*__*stem*_ values, *R* ^2^ was 0.76 for *T*_s_–*T*_a_, 0.79 for WDI_92 and WDI_99.8, and 0.80 for WDI_t. All these correlations were highly significant (*P* ≤ 0.001). When the data set was considered all dates confounded, linear regression between *ψ*_*H*__*stem*_ and *T*_s_–*T*_a_ showed less significant correlation (Fig. 6M; *P* ≤ 0.01; *R* ^2^ = 0.19) than regression between *ψ*_*H*__*stem,*_ and WDI_qr (Fig. 6N–O; *P* ≤ 0.001; *R* ^2^ = 0.52 to 0.57) while correlation between *ψ*_*H*__*stem,*_ and WDI_t was no more significant (Fig. 6P; *R* ^2^ = 0.06).

## Discussion

Remotely sensed images were acquired for different dates and irrigation regimes at an altitude of 300 m, and the whole orchard trial was covered by one or two TIR images with a 30- to 40-cm pixel size. This intermediate image resolution made it possible to phenotype the whole experimental plot using a series of snapshots and to work at the tree level, with individuals distinguishable from each other (Berni *et al.*, 2009).

### Limits of T_s_–T_a_ as a water stress indicator

The canopy surface temperature considered for each tree was an average of numerous leaf temperatures, located within a representative canopy central zone, thus buffering the effects of leaf angular distribution (Jones *et al.*, 2009). Indeed it is often preferable to use thermal measurement of the canopy surface instead of particular leaves (Maes and Steppe, 2012).

When the experiment dates were considered separately, the mean *T*_s_–*T*_a_ values of WS trees compared to those of WW trees were consistent with the irrigation regime applied and showed a significantly higher canopy temperature when water was withheld. Differences between mean *T*_s_–*T*_a_ of WW and WS trees were consistent with the diminution of soil water potential *ψ*_*s*_, thus enabling discrimination between water-constrained and well-irrigated trees.

Nevertheless, as described in the literature (Maes and Steppe, 2012), our results also highlighted difficulties in the interpretation of *T*_s_–*T*_a_ variations across time. Indeed, surface temperatures for date 1 were higher than those observed later, particularly in WW trees. This probably resulted from the lower evaporative demand for date 1 (VPD = 1.47 kPa) compared to dates 2 and 3 (VPD = 2.51 and 2.41 kPa, respectively), while *T*_s_–*T*_a_ was lower for dates 2 and 3 in WW trees, as a likely consequence of higher transpiration rates. As the effect of soil drought was opposite, with a 6°C temperature rise in WS compared to WW trees for dates 2 and 3, both effects could compensate, explaining why *T*_s_–*T*_a_ revealed no significant differences between dates in the WS treatment. As a consequence, this sole stress indicator is not reliable for temporal analysis of soil drought response.

Another point described in the literature concerns the proportion of soil viewed through the vegetation which can be a source of error in interpretation of *T*_s_–*T*_a_ (Jackson *et al.*, 1981; Moran *et al.*, 1994). Tree values of *T*_s_–*T*_a_ were averaged inside a buffer zone of 60cm radius. Presence of soil and mixed pixels inside this buffer zone, due to genotypic differences in vegetation cover fraction and structure, can create a bias in estimation of water status for the smallest trees or in those whose architecture is porous. As the raw *T*_s_–*T*_a_ variable is not corrected from soil temperature, this can be a source of error in phenotypic analyses (White *et al.*, 2012).

### Limits of the WDI theoretical approach

In our conditions, applying the theoretical method for delineating the VIT scatterplot did not yield satisfactory results. For the three dates of the experiment, the theoretical envelopes overlapped either the left or the right border of the VIT scatterplot. Computation of WDI_t produced values that were underestimated and out of the expected range for date 3. Although a theoretical approach enabled relative comparisons of WS and WW trees for a given date, comparisons between dates yielded contradictory results.

These difficulties may be explained by heavy model implementation and numerous sources of error caused by estimation of some parameters, especially in the context of high-throughput phenotyping. The energy transfer between soil and vegetation can differ between the full-cover fraction of a field crop and the partial-cover vegetation of an orchard. Heilman *et al.* (1996) suggested that a large variation in the soil energy balance (vertices 3 and 4, Fig. 1) can result from its sensitivity to the wind speed *u*, the aerodynamic resistance *r*_*a*_, and the surface temperature *T*_*s*_ when crops are trellised and/or organized in rows. This is typically the case in an orchard or a vineyard (Galleguillos *et al.*, 2011). Moreover, the net radiation *R*_*n*_, which depends on the albedo *a* and emissivity *ε*, is sensitive to the surface composition and row orientation (Van de Griend and Owe, 1993; Zarco-Tejada *et al.*, 2005). Another limit to the application of the theoretical determination of WDI for the purpose of phenotyping is the architectural variability of the vegetation cover at the individual plant level. Indeed, the trees differed in height (1.5 to 3.5 m) and in vegetation density (0 < NDVI < 0.18). One way to improve the theoretical energy balance model for a heterogeneous population would be to parameterize this model for the most contrasted genotypes in response to water constraints. Nevertheless, implementing the theoretical model would require a previous identification of extreme genotypes of the population before phenotyping and measurements of the canopy physical characteristics (the aerodynamic and stomatal resistance, *r*_*a*_ and *r*_*s*_, the height, *h*, the net radiation, *R*_*n*_, and the soil heat flux, *G*).

### Contribution of the statistical approach

Quantile regression enabled scatterplot delineation at each of the three dates of experimentation. Our results showed that the quantile percentage of the scatterplot used to define the left and right boundaries influenced the WDI values. A reduction in the scatterplot percentage resulted in an increase in the range of variation of WDI values and a significant increase in the mean WDI values for trees submitted to water constraints (Table 2). These variations can mainly be attributed to divergence of the right limits at the upper right side of the scatterplot. Like WDI_t and *T*_s_–*T*_a_, WDI_qr enabled differentiation between WW and WS trees when a contrasting irrigation regime was applied at a given date (dates 2 and 3), whatever the quantile applied. Moreover, use of WDI_qr as a stress indicator in WS trees produced mean tree values that were consistent with evolution of *ψ*_*s*_ for the three dates (Table 1) compared to values of WDI_t and *T*_s_–*T*_a_.

Assessing the WDI_qr values in more depth would require the determination of the actual to maximal evapotranspiration ratio for each tree of the population in the orchard, which seems unrealistic. Nevertheless, the statistical method used to delineate the VIT trapezoid yielded typical WDI values for the trees, in the range of 0 to 1, at least for the quantile panel tested. Moreover, quantile regression was relatively easier to implement in regard to the theoretical method.

### Water stress indicator and sensitivity to environmental conditions

For apple trees, *ψ*_*H*__*stem*_ constitutes a robust estimator of plant water status (Naor 2006; Doltra *et al.*, 2007). In our case, on dates 2 and 3, which presented contrasting hydric conditions, the stem water potential was well correlated with the different water stress indicators (*T*_s_–*T*_a_, WDI_92, WDI_99.8, and WDI_t). However as described in the previous section, *T*_s_–*T*_a_ and WDI_t values were inconsistent with soil drought for some dates. This resulted in a poor correlation of these indicators with stem water potential when all dates were confounded (Fig. 6). Concerning WDI_qr, although variation in the quantile levels could impact its values, the quality of the relationship between *ψ*_*H*__*stem*_ and WDI_qr was little affected, considering dates 2 and 3 separately. For a temporal comparison WDI_qr thus appeared as the most robust index as shown by the highest correlation with *ψ*_*H*__*stem*_ in comparison to *T*_s_–*T*_a_ and WDI_t.

Stable WDI_qr values computed for the WS trees for dates 2 and 3 were not fully consistent with the variations of *ψ*_*H*__*stem*_, which indicated increasing water stress in the WS trees between these two dates, and revealed a possible underestimation of the water stress by WDI_qr at date 3. One possible explanation is the time of image acquisition, which was slightly earlier in the day on date 3 (9:20 GMT) than on date 2 (10:00 GMT), i.e. at an earlier phase of daily stomatal regulation for date 3, while subsequent measurement of the stem water potential reflected the daily minimum *ψ*_*H*__*stem*_ in both cases. Another possibility is that the development of the fruit sink strength increasingly stimulated photosynthetic activity and carbon acquisition, allowing a concomitant continuation of transpiration and a decrease in *ψ*_*H*__*stem*_; low midday stem water potentials in apple trees, decreasing with increasing crop loads, have been shown by Naor *et al.* (2008). This can create an upper limit on the highest WDI values observed, even after water stress was established. A third possibility is the influence of a lower soil water potential *ψ*_*s*_ at date 3 than at date 2 (see Table 1), producing a shift in *ψ*_*H*__*stem*_ toward more negative values. These three hypotheses are non-exclusive.

## Conclusion

This study confirmed that thermal infrared imagery contributes interestingly to the phenotyping of crop response to water stress. Based on multispectral images acquired, the VIT concept constitutes a relevant approach that takes the environmental conditions and vegetation cover fraction into account. Moreover it allows a characterization of the water status of a whole tree population with heterogeneous architectural traits and an irregular cover fraction. To avoid the difficulties of parameterization of the theoretical WDI model and allow more operational phenotyping, a simplified statistical approach based on quantile regression has been proposed to delineate the boundaries of the VIT scatterplot needed for WDI computation. Regardless of the percentage of the scatterplot used (from 92 to 99.8%) to delineate the VIT trapezoid, the resulting WDI_qr was well correlated to the stem water potential.

Unlike with a WDI theoretical approach or *T*_s_–*T*_a_, a WDI statistical approach produced values consistent with temporal variations of water status. The range of WDI_qr values (0.20 to 0.65) indicates its sensitivity to water constraint, and a next step will be exploring the genetic variability of this response within the tree population. Further investigations will also attempt to evaluate the daily dynamics of stomatal regulation of the 122 hybrids in response to water constraints through yielding successive images during the same day and assessing the potential of a WDI statistical approach at this time scale. This analysis could contribute to discriminating the genotypic stomatal behaviours (isohydric vs anisohydric). Thanks to its relatively simple implementation, the quantile regression approach shows promise as an efficient tool for thermal imagery and use in phenotyping studies.

## Funding

The PhD scholarship of Nicolas Virlet was granted by Montpellier SupAgro and the Languedoc Roussillon Region.
